# Model prediction of a Kunitz-type trypsin inhibitor protein from seeds of Acacia nilotica L. with strong antimicrobial and insecticidal activity

**DOI:** 10.3906/biy-2002-20

**Published:** 2020-08-19

**Authors:** Sohaib MEHMOOD, Muhammad IMRAN, Arslan ALI, Aisha MUNAWAR, Binish KHALIQ, Farzeen ANWAR, Qamar SAEED, Friedrich BUCK, Saber HUSSAIN, Ahsan SAEED, Muhammad YASIN ASHRAF, Ahmed AKREM

**Affiliations:** 1 Botany Division, Institute of Pure and Applied Biology, Bahauddin Zakariya University, Multan Pakistan; 2 Forman Christian College (A Chartered University), Lahore Pakistan; 3 Dr. Panjwani Center for Molecular Medicine and Drug Research, International Center for Chemical and Biological Sciences, University of Karachi, Karachi Pakistan; 4 Institute of Chemical Sciences, University of Engineering and Technology, Lahore Pakistan; 5 Department of Entomology, Bahauddin Zakariya University, Multan Pakistan; 6 Institute of Clinical Chemistry, University Hospital Hamburg-Eppendrof, Hamburg Germany; 7 Institutes of Molecular Biology and Biotechnology, University of Lahore, Lahore Pakistan

**Keywords:** * Acacia nilotica*, Trypsin Inhibitor, antimicrobial, entomotoxin, *Tribolium castaneum*, *Sitophilus oryzae*

## Abstract

A Kunitz-type trypsin inhibitor protein has been purified and characterized from seeds of *Acacia nilotica *L. LC-MS/MS analysis of *Acacia nilotica* trypsin inhibitor (*An*TI) provided the N-terminal fragment of 11 amino acids which yielded 100% identity with already reported Kunitz-type trypsin inhibitor protein of *Acacia confusa* (*Ac*TI) in UniProtKB database search. SDS-PAGE showed a single band of ~21 kDa under nonreduced condition and appearance of a daughter band (17 kDa) in the presence of *β*-mercaptoethanol indicating the presence of interchain disulfide linkage typical for Kunitz-type trypsin inhibitors. *An*TI was purified from seed extract by using a combination of anion exchange and gel filtration chromatography. Since *An*TI showed maximum homology with *Ac*TI, a molecular structure of *Ac*TI was predicted which showed highly *β*-sheeted molecular conformation similar to crystallographic structure of* Enterolobium contortisiliquum* trypsin inhibitor (*Ec*TI). *An*TI (20 µg) produces significant population inhibition against different human pathogenic bacteria along strong antifungal activity (50 µg). Entomotoxin potential of *An*TI was evaluated against two stored grain insect pests *Tribolium castaneum *(Herbst) (Tenebrionidae: Coleoptera) and *Sitophilus oryzae *(Linnaeus) (Curculionidae: Coleoptera). Statistically significant mortality of *T. castaneum* adults was observed at 1.5 mg after 15 days in comparison to control. Additionally, number of total eggs, larvae, pupae, adults, and their male/female ratio were also severely reduced in comparison to control. Similarly, two generation progeny of *S. oryzae *was studied after mixing *An*TI with rice kernels. Mean percent mortality of adult population was significantly higher after 9 days of exposure in comparison to control group. *An*TI significantly reduced the F1 generation while little mortality was observed for F2 generation. Exploration of such potent molecules is the prerequisite of our time regarding the anticipation of postantibiotic era and the development of insect resistance against chemical pesticides.

## 1. Introduction

Seeds are the vehicles for continuity of next generation and contain various proteinaceous enzyme inhibitors such as amylase inhibitors and proteinase/protease inhibitors (PIs) (Furstenberg-Hagg et al., 2013). The protease inhibitors in different organs of the plants are expressed in response to the abiotic stresses as well as to pathogens and insects (Jamal et al., 2013; Yamchi et al., 2018). Trypsin inhibitors (TIs), one of the major classes of serine protease inhibitors, are classified in two families according to their molecular size as Kunitz-type trypsin inhibitors (KTIs; 21 kDa) and Bowman–Birk trypsin inhibitors (BBTIs; 8 kDa) (Rao and Suresh 2007; Armstrong et al., 2013; Bendre et al., 2018). Kunitz-type trypsin inhibitor was first characterized from *Enterolobium contortisiliquum* (*Ec*TI) (Batista et al., 1996). Plant-based KTIs have shown inhibition of trypsin or chymotrypsin along other serine proteinases such as subtilisin and elastase (Revina et al., 2004; Sumikawa et al., 2006). Some individual KTIs typically have shown more specific activities in comparison to those that inhibit cysteine or aspartic proteinases (Heibges et al., 2003). 

Kunitz-type trypsin inhibitors are also found in all *Leguminosae* subfamilies including *Mimosoideae*,* Caesalpinioideae*, and *Papilionoideae* (Furstenberg-Hagg et al., 2013)*.* *Acacia nilotica* (babool) is one of the common species of the genus *Acacia*. For a long time the *Acacia* seeds have been used as a source of food for the native people of Australia and they have substantial economic weight due to its widespread accessibility by agriculture and wild harvests (Ahmed and Johnson, 2000). *A. nilotica* has been used for the therapy of ear and eye tumors (Ahmad et al., 2008) in addition to curing diarrhea, leprosy, dysentery, ulcer, cancers, and diabetes (Aliyu, 2006). Commercially, the seeds of *Acacia* have been used as a flavoring agent in dietary products and in beverages as an ingredient (Maslin and McDonald, 2006). 

KTIs are widely studied because of their large number of biological properties. The biochemical characterization of many individual plant KTIs has already been done, e.g., in potato (*Solanum tuberosum*), analysis of a large KTI family has shown surprising sequence variations, including many nonsynonymous substitutions which translated into functional diversity (Heibges et al., 2003; Major and Constabel, 2008). Plant KTIs possess a very broad range of protease targets and thus have deleterious effects on phytophagous pests and pathogens. Some plant KTIs have shown antimicrobial activity, presumably through inhibition of microbial proteinases (Kim et al., 2005; Park et al., 2005; Bendre et al., 2018). Therefore, in vitro assays are necessary for the confirmation of antimicrobial potential of these KTIs parallel to primary sequence similarities. Thus, our study aimed at the identification and functional characterization of KTI from the seeds of *A. nilotica* (*An*TI) which have the amino acids sequence homology with the already reported Kunitz-type trypsin inhibitor from *Acacia confusa* (Eriksson et al., 1993). 

## 2. Materials and methods

### 2.1. Plant material

Seeds of *Acacia nilotica* were taken from the Botanical garden, Bahauddin Zakariya University, Multan, Pakistan and were kept at room temperature. 

### 2.2. Isolation and purification of trypsin inhibitor protein

Twenty gram seeds of *A. nilotica* were in 100 mL Tris buffer (100 mM, pH 7.0) for 12 h at 4 oC and then homogenized with blender. The extract was centrifuged (Heraeus Thermo Labofuge 200) at 5000 rpm for 20 min and supernatant was subjected to Hi-Trap Q-FF–anion exchange chromatography Column (0.7 × 2.5 cm) (GE Healthcare Life Sciences, 17505301). The buffer solutions and protein extract were filtered by passing through 0.22 µm using a 10 mL syringe. Two cycles were executed and for each cycle (run) absorbance peak was recorded in the form of chromatogram. The column was preequiliberated with 5 mL of Tris buffer (100 mM, pH 7.0) using ÄKTA pure protein purification system (GE Healthcare Life Sciences) at a flow rate of 1.0 mL/min. Clear protein extract (5 mL) was concentrated to 1 mL by using a concentrator (3 kDa MWCO, Amicon) and then loaded on anion exchange column for one cycle. Linear gradient (0-500 mM NaCl) of buffer B (0.1 M Tris, pH 7.0) was used for protein elution at flow rate of 1.0 mL/min. Highly purified trypsin inhibitor bands shown by SDS-PAGE were pooled together and dialyzed against buffer A (100 mM Tris, pH 7.0) containing 150 mM NaCl. For the next purification step, the pooled sample was loaded onto a gel filtration column Superdex 200 Increase 10/300 GL (Catalog no. 28990944; 10 mm × 300 mm) preequiliberated with Tris buffer (100 mM, pH 7.0) containing 150 mM NaCl. After size exclusion chromatography (SEC), the protein fractions were collected, properly labelled, and stored at 4 °C until SDS-PAGE analysis. In all purification steps, absorbance of the proteins was monitored at 280 nm.

### 2.3. SDS-PAGE and molecular mass determination

Protein quantification was done using NanoDrop method (Desjardins and Conklin, 2010) and SDS-PAGE (12%) was run according to the standard protocol (Laemmli 1970) to analyze the protein banding pattern. The electrophoretic mobilization of protein bands was compared with standard protein marker (PageRularTM Unstained Protein Ladder, 10-200 kDa; Catalog No. BM 201) to find out the relative molecular weight of the protein. 

### 2.4. Trypsin inhibition assay

Estimation of the bovine trypsin inhibition potential of crude* An*TI and corresponding protein fractions of each purification step was done according to Erlanger et al., (1961) by using Nα-benzoyl-DL-arginine-*p*-nitroanilide (BA*p*NA) as substrate. For inhibitory assay, trypsin (2.5 µg) solubilized in 1 mM HCl, was incubated with *An*TI obtained from each purification step in 50 mM Tris–HCl, pH 7.5. Following an incubation (37 °C) period of 15 min, the reaction was started by adding 500 µL of 1.25 mM BA*p*NA. After 30 min, the reaction was stopped by addition of 250 µL of 30% (v/v) acetic acid solution. Substrate hydrolysis was observed by recording absorbance at 410 nm. One trypsin inhibitory activity was defined as a decrease of 0.01 units of absorbance at 410 nm at 37 °C.

### 2.5. LC-MS/MS mass spectrometry

Stained protein bands were cut out from the gel and reduced by using dithiothreitol (10 mM, 55 °C, and 30 min). Through trypsin enzyme, the protein bands were digested overnight as per standard protocol (Hewick et al., 1981). The digested pieces of gel were dried in a vacuum concentrator after extraction using 50% (v/v) acetonitrile/5% (v/v) formic acid solution. The measurements of LC-MS/MS were made by injecting samples onto a nano liquid chromatography system (Dionex Ultimate 3000) coupled to an electrospray ionization ion source of an Orbitrap mass spectrometer (Orbitrap FusionTM Germany). The sample was loaded on a trapping column (Acclaim PepMap C18 75 µm × 2 cm; buffer A: 0.1% formic acid in H_2_O; buffer B: 0.1% formic acid in acetonitrile) with 2% buffer B and peptides were eluted (300 nl/min) against the separation column (Acclaim PepMap 100, C18 75 µm × 25 cm). The analysis of LC-MS/MS was made in data-dependent acquisition mode (DDA) and raw data of LC-MS was analyzed using Proteome Discoverer 2.0 (Thermo Fisher Scientific, Germany). For the identification of protein, MS/MS spectra were searched with Sequest HT against the *Arabidopsis* and UniProtKB databases with the following parameters. Precursor mass tolerance and fragment mass tolerance were set to 10 ppm and 0.5 Da, respectively. Two missed cleavages were allowed, while variable modification of methionine oxidation and fixed modification of carboxymethyl at cysteines were also used. 

### 2.6. Model prediction

*An*TI mass spectrometric residual sequence showed maximum homology with primary sequence of* A. confusa *trypsin inhibitor (*Ac*TI). The primary sequence of *Ac*TI was subjected to model building in Swiss-Model server (Biasini et al., 2014) taking the coordinate information of *Enterolobium contortisiliquum* trypsin inhibitor protein (*Ec*TI). Model was built based on the target-template (PDB ID: 4J2K) alignment using ProMod (Benkert et al., 2010). The conserved coordinates between template and target were copied to the model. A fragment library and side chains were used to remodel the insertions and deletions. The resulting model geometry was regularized by using a force field. The quality of the model was determined by Ramachandran plot by using ProCheck online server1http://services.mbi.ucla.edu/PROCHECK/ (Laskowski et al., 2006). The structural details were depicted as cartoon models by using PyMOL2DeLano WL (2002). The PyMOL molecular graphics system. http://www pymol org.

### 2.7. Antibacterial activity

Antibacterial activity of *An*TI was assessed by microplate assay (Amsterdam 1996) against the different pathogenic bacteria such as *Bacillus subtilis*, *Escherichia coli*,* Pseudomonas aeruginosa*,* Staphylococcus aureus*, and* Xanthomonas oryzae*. Luria-Bertani (LB) growth medium was used for the preparation of bacterial culture which was inoculated with 100 μL bacterial cells and incubated at 37 °C overnight. 100 µL of fresh bacterial culture was treated with 100 µL of *An*TI containing different concentrations (5, 10, 15, 20, 25, and 30 μg). Antibiotic (Calamox, Bosch) was used as a positive control and Tris buffer (100 mM, pH 7.0) as a negative control. Five replicates of all the treatments were made and absorbance was recorded at 600 nm on hourly basis up to 8 h of incubation at 37 °C. Data have been presented as Excel graphs after calculating the means, standard deviations, and errors. After careful observation of the microbial growth inhibition as a result of different *An*TI concentrations, two treatments (15 and 20 μg) were further assessed against pathogens by using the standard procedure of Kirby–Bauer disc diffusion method (Boyle et al., 1973). 

### 2.8. Antifungal activity

Two fungal species *Fusarium oxysporum* (FCBP-PTF-866) and *Aspergillus niger* (FCBP-PTF-706) were purchased from the First Culture Bank of Pakistan, University of Punjab, Lahore, Pakistan. The fungal cultures were stored at 4 °C and were propagated on YPSA medium. Mature conidia of both fungal strains were prepared from mature fungal cultures grown in YPSA plates at 25 °C. Fifteen milliliters of prechilled Tris buffer (100 mM, pH 7.0) was added to the petri dishes and stirred gently for 5 min. Asexual spores were numbered at 400× magnification using hemocytometer (NeubauerHausser Bright-Line; Catalogue No. 3100) and adjusted to a standard concentration of 1 × 10^5^ cell mL^-1^. A range of *An*TI concentrations (30, 40, 50, 60, and 70 µg/100 µL/well) were tested against condial growth in 200 µL 96-well titre plates after incubating at 25 °C. Tris buffer alone was used as negative control while fungicide Topsin® 4.5 FL was used as positive control. Absorbance (600 nm) data were measured at 0, 24, and 48 h of postincubation. Experiment was carried out with five replicates while the mean and other statistical values were calculated. Results are presented in graphical form to express the inhibition pattern of conidial germination. Further, 50 µg/disc *An*TI concentration was reevaluated against the two fungi by using disc diffusion assay similar to antibacterial experiment. The sterilized discs (15 mm) of filter paper were placed at equidistance from the center of the petri plate. Fungicide Topsin® 4.5 FL (10 µL) was taken as a positive control and Tris buffer (60 µL) was taken as a negative control while 50 µg/disc *An*TI was used for fungal growth inhibition. The fungus culture was placed carefully in the center of the petri plate and kept in an incubator at 30 °C. After 48 h of incubation, the fungal growth was analyzed and the results were recorded.

### 2.9. Insecticidal activity

Two stored grain insect pests (*Tribolium castaneum *and* Sitophilus oryzae*) were chosen to check the insecticidal potential of *An*TI. The insect cultures were maintained as per standard protocol (Cardona et al., 1989) at 30 °C with 70% relative humidity. In first experiment, 150 g wheat flour was treated with three concentrations of *An*TI (0.5, 1.0, and 1.5 mg) and control (Tris-base, 100 mM, pH 7.0). Each concentration was replicated into 5 times for wheat flour (20 mg each) and five pairs of *T. castaneum *were released in each experimental unit. After 15 days, the adults were removed and data were recorded weekly for number of eggs, larvae, pupae, and next generation’s adults. 

In another experiment, similar *An*TI concentrations were sprayed on 100 g of rice kernels to evaluate the entomotoxicity against *S. oryzae* population. Each dose was again replicated five times and experiment was performed in a glass jar with five pairs of insects in each replicate. Survival data were recorded after every 3 days up to 9 days and compared with the control group. The surviving individuals were left for the next progeny to see the protein effectivity on the lifespan of rice weevil after 35 (F1 generation) and 65 (F2 generation) days. 

### 2.10. Statistical analysis

By using the analysis of variance and means, the data of *T. castaneum *and *S. oryzae *were examined and measured by Tukey’s test (HSD) using the software Statistics 8.1 (Analytical Software, 2005).

## 3. Results 

### 3.1. Gel analysis of AnTI

**Figure 1:**
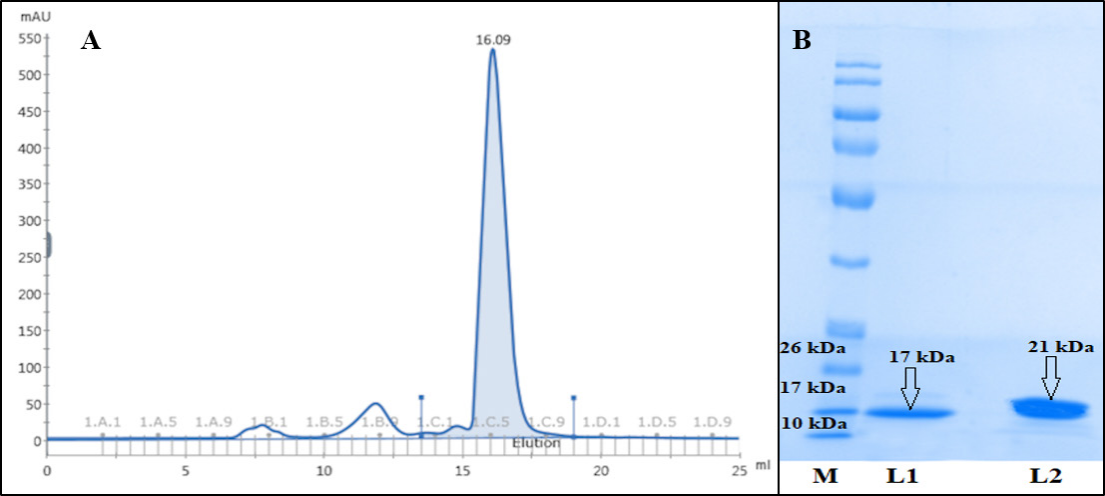
SEC chromatogram and the corresponding gel show the purification of *An*TI. A is the size exclusion chromatogram of *An*TI (Superdex 200 Increase SEC). B shows the purity of *An*TI on SDS-PAGE under both reduced and nonreduced conditions. M: the standard protein ladder, L1: reducing condition (17 kDa), L2: nonreducing condition (21 kDa).

A thorough reproducible purification strategy was developed for the purification of *An*TI as indicated in Table. Twenty grams of seeds produced almost 291 mg total protein in crude extract which reduced to 74 mg after anion chromatography with percent yield reduction of more than 70%. Gel filtration (Figure 1A) at the end yielded 12.94 mg total purified *An*TI with 1.37 purification fold. Highly purified *An*TI showed a specific activity of 0.056 × 10^5^ TIU/mg and a yield of 6.09% as presented in Table. Purified *An*TI showed 21 kDa band under nonreduced condition and the corresponding reduced band of approximately 17 kDa (Figure 1B); indicating the presence of interchain disulfide linkage which is typical for this class of proteins. 

**Table:** Purification of A. nilotica trypsin inhibitor AnTI.

Steps	Total protein (mg)	Total activity(TIU) × 10^5^	Specific activity(TIU) × 10^5^	Yield(%)	Purification(fold)
Crude extract	290.95	11.97	0.0411	100	1
Anion Exchange-Hi trap Q FF column	73.84	3.2	0.0433	26.73	1.05
SEC Superdex increase 200- 10/300 GL	12.94	0.73	0.0564	6.09	1.37

### 3.2. Protein identification by LC-MS/MS spectrometry

**Figure 2:**
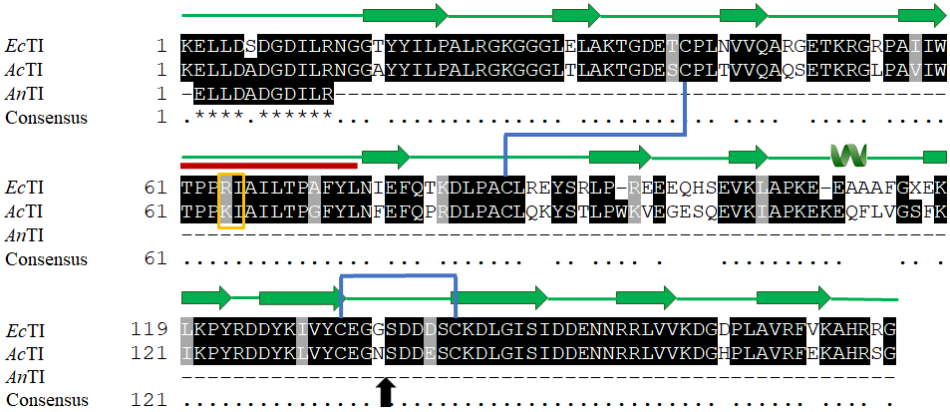
Multiple sequence alignment of MS generated *An*TI residues (11) with other trypsin inhibitors. *Enterolobium contortisiliquum* (*Ec*TI; P86451) and *Acacia confusa* (*Ac*TI; P24924) showed 100% sequence identity. Secondary elements including single α-helix and multiple β-sheets of *Ec*TI are indicated at the top of sequence alignment; blue lines indicate highly conserve disulfide bridges; red bar indicates reactive loop, and yellow box covers reactive residues. Black arrow indicates the point of detachment of the two chains (α & β) in the presence of β-mercaptoethanol. Black shade represents the identity while gray is dissimilarity.

The reduced 17 kDa band of *An*TI was subjected to mass spectrometry which yielded a fragment of 11 residues. The tryptic peptide sequence ELLDADGDILR was BLAST in uniProtKB online server3www.uniprot.org  (Jain et al., 2009) which showed 100% sequence identity with already reported trypsin inhibitors of *Acacia confusa* (*Ac*TI) and other different plants. The fragmented sequence of *An*TI was aligned with primary sequences of trypsin inhibitors of *Acacia confusa *and *Enterolobium contortisiliquum *to highlight the secondary elements and the presence of disulfide linkages (Figure 2). 

### 3.3. Predicted AcTI model

**Figure 3:**
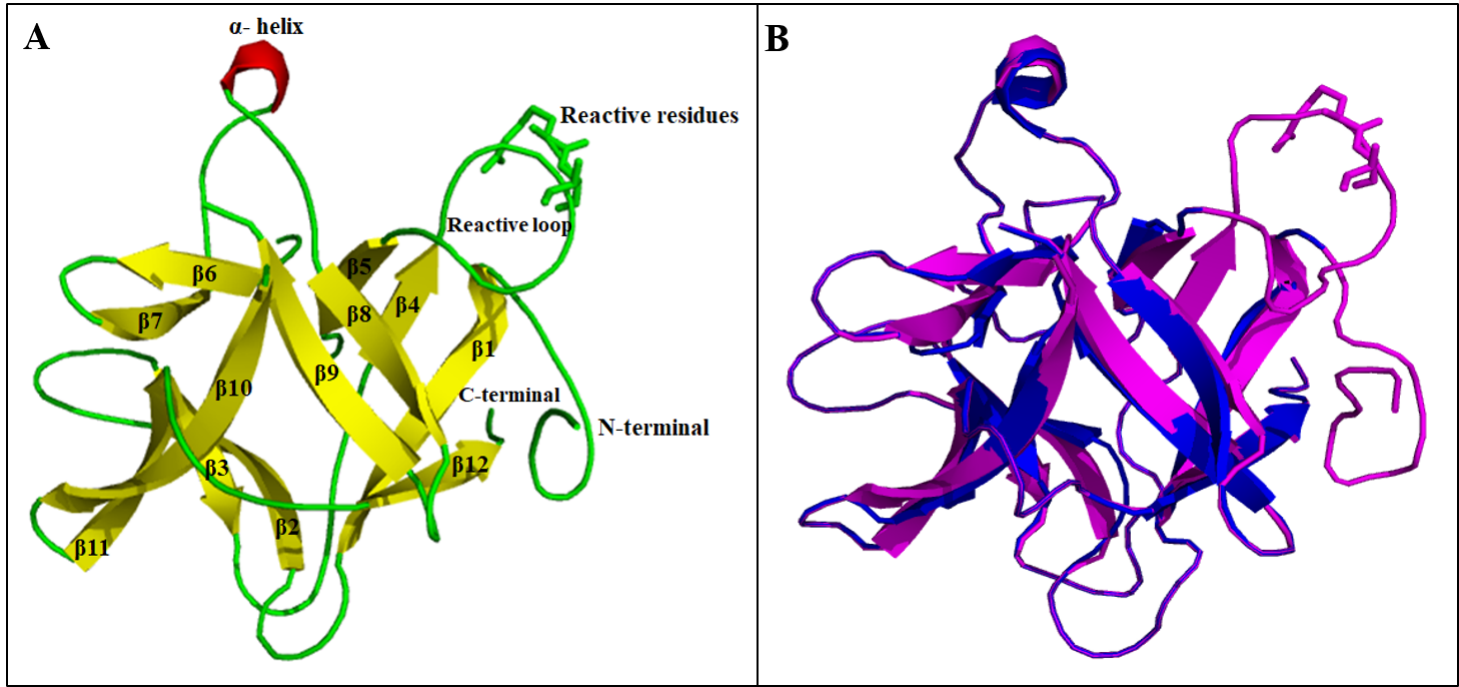
Cartoon diagrams showing the structural features of *A. confusa* trypsin inhibitor (*Ac*TI). A is the predicted 3D ribbon model and shows the presence of single surface-based α-helix, 12 β-sheets, 12 loops, reactive residues Arg (64) & Ile (65) as well as N and C terminii. B is the structural alignment of *Ac*TI model (magenta) with *Ec*TI (PDB ID: 4J2K; blue) which indicates a highly homologous molecular conformation with RMSD value of 0.69.

*An*TI fragment (11 residues) showed 100% homology with *Ac*TI; therefore, primary sequence of *Ac*TI was used for the calculation of the predicted model. Predicted 3D ribbon model of *Ac*TI was thoroughly analyzed and overall structural features are shown as cartoon models (Figure 3). The model was verified through ProCheck online server and generated Ramachandran plot showed 89.3% residues in most favored region, 8.6% in additional allowed region, 0.7% in generously allowed region and only 1.4% in disallowed region. Structural alignment of *Ac*TI (magenta) predicted structure with *Ec*TI (PDB ID: 4J2K) crystallographic conformation (blue) indicated a highly homologous molecular conformation with RMSD value of 0.69. 

### 3.4. Assay of antibacterial activity

**Figure 4:**
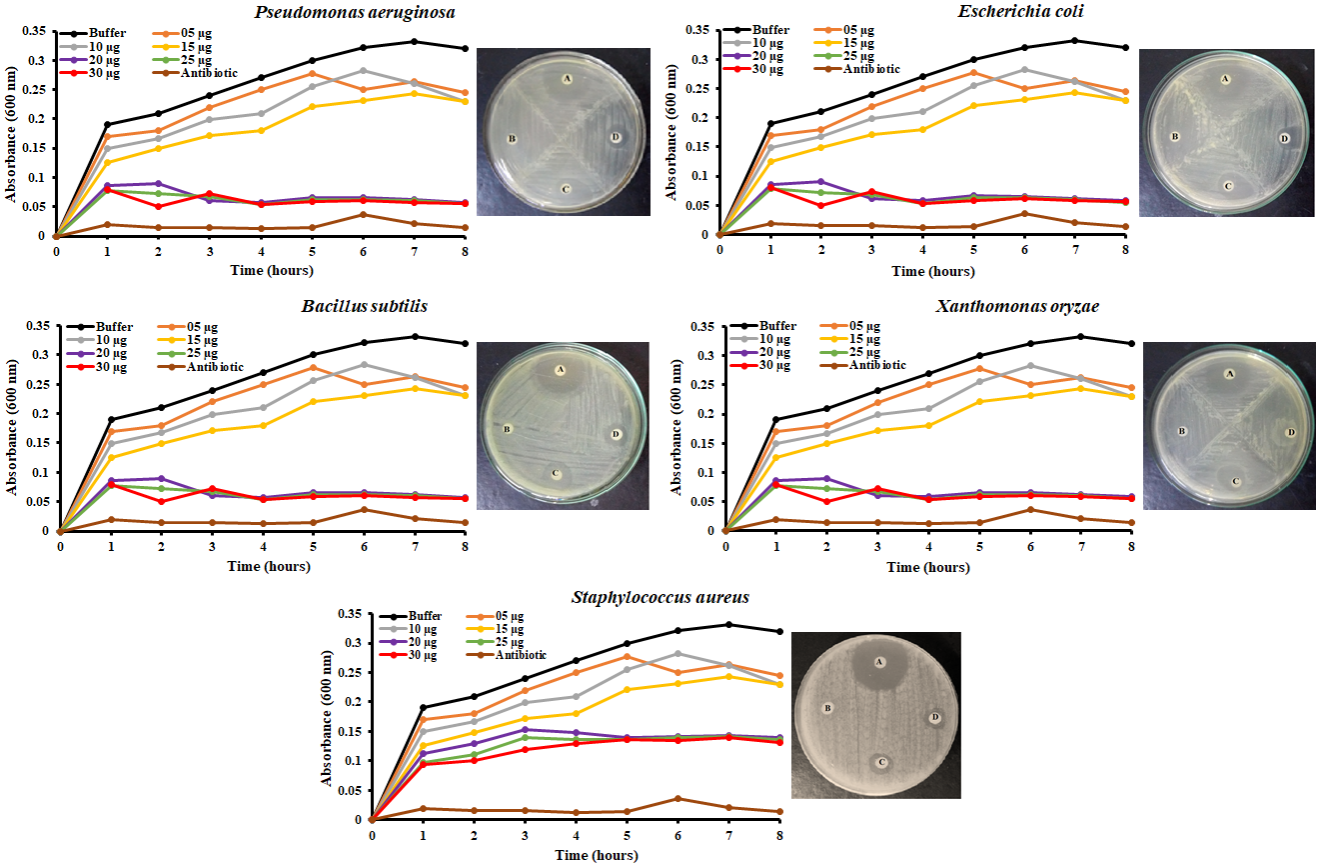
Antibacterial assays of various *An*TI concentrations showing growth inhibition of different pathogenic bacterial strains. Microplate assay showed time barred growth inhibition of all gram-positive and gram-negative pathogens. Significant inhibition has been observed for 20 μg *An*TI treatment in comparison to lower values. Additionally, the results were cross-verified through disc diffusion assay showing the inhibition of bacterial growth as a result of 15 and 20 μg AnTI per disc. A: Calamox (5 μg/disc); B: Buffer (100 mM Tris buffer; pH 7.0); C: Concentrated protein (20 μg/disc); D: Diluted protein (15 μg/disc).

The antibacterial effect of the purified *An*TI was checked against five human pathogenic bacterial species. Different concentrations produced varied levels of bacterial growth inhibition. More than 50% bacterial population inhibition was observed for 20 µg of *An*TI in comparison to control at 8 h of incubation. Two higher concentrations (25 and 30 µg) did not produce any further significant improvement of bacterial killing in comparison to 20 µg. Absorbance values were recorded at 600 nm on hourly basis and the data are presented in Figure 4. Additionally, two concentrations (15 and 20 µg) were again tested on petri dishes against all bacterial strains and similar degrees of population inhibition were observed as indicated by the titer assay. *S. aureus* was observed to have relatively mild effects of growth inhibition as compared to other bacterial strains in response to *An*TI treatments as shown in Figure 4.

### 3.5. Assay of antifungal activity

**Figure 5:**
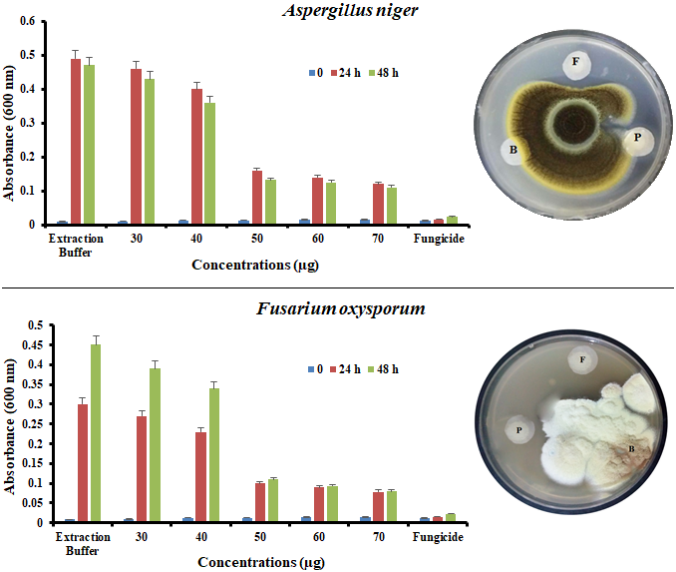
Antifungal assays of various *An*TI concentrations are showing growth inhibition of different phytopathogenic fungal strains. Significant conidial inhibition was observed by 50 μg and both fungi responded to *An*TI in similar pattern with maximum inhibition started between 24–36 h of incubation. 50 μg of *An*TI was further checked through disc diffusion method and exhibited strong inhibition of fungal mycelia. F: Topsin® fungicide (10 μL); B: Buffer (100 mM. Tris buffer; pH 7.0); P: *An*TI (50 μg/disc).

The antifungal activity of the *An*TI was checked against *Aspergillus niger *and *Fusarium oxysporum*. Conidial germination and mycelial growth in response to different *An*TI concentrations was observed for 48 h of incubation. *An*TI at a concentration of 50 µg/well strongly inhibited the mycelial growth of both fungal strains. Control showed maximum growth of mycelia while fungicide completely suppressed the conidial growth. *An*TI concentrations higher than 50 µg/well did not further improve the fungal inhibition. Additionally, the same 50 µg *An*TI concentration showed strong mycelial growth inhibition of *Aspergillus niger *and *Fusarium oxysporum* in disc diffusion method (Figure 5). 

### 3.6. Insecticidal efficiency of AnTI against T. castaneum

**Figure 6:**
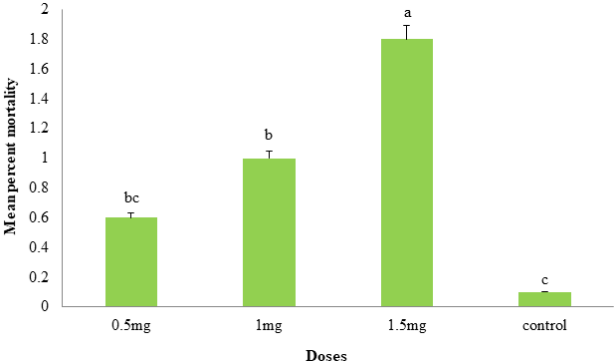
Mean percent mortality (±SE) of *Tribolium castaneum* in response to three doses of *An*TI protein. Three doses of *An*TI (0.5, 1.0, and 1.5 mg) were exposed with 5 pairs (male and female) of *T. castaneum* adults. Statistically significant mean percent mortality was observed at 1.5 mg after 15 days in comparison to control (0.1 M Tris buffer; pH 7.0).

Adults of *T. castaneum* were exposed to three doses of *An*TI (0.5, 1.0, 1.5 mg) for 15 days and produced mean mortalities of 0.6 ± 0.24, 1.0 ± 0.31, 1.8 ± 0.2, respectively. Two treatments (1.0 & 1.5 mg) produced significant mortalities, while 0.5 mg produced nonsignificant population reduction in comparison to control (Figure 6).

### 3.7. Effect of AnTI on different life stages of T. castaneum

Different life parameters of *T. castaneum* progeny were observed after exposure to *An*TI. Number of total eggs, larvae, pupae, adults, and male/female ratios were calculated and significant population reductions were observed for all parameters in comparison to control group. However, the treatments produced nonsignificant mortalities in comparison to each other.

#### 3.7.1. Egg stage

**Figure 7:**
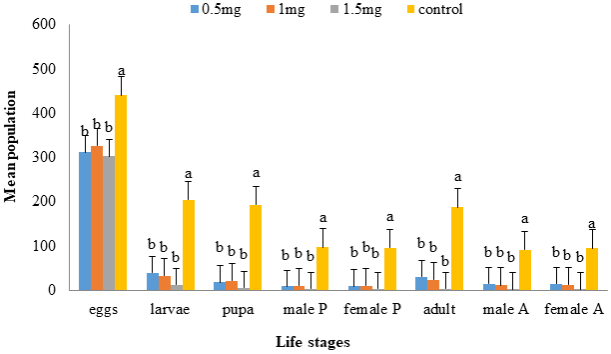
Effect of *An*TI protein on different life stages of *T. castaneum*. One generation progeny of *T. castaneum* was studied after exposure of *An*TI mixed with flour. *An*TI produced significant reductions in all life parameters including number of total eggs, larvae, pupae, adults, and their male/female ratio in comparison to control group.

At statistically significant difference, minimum number of eggs were laid, i.e. 303 ± 9.94 at maximum dose of 1.5 mg followed by 1.0 and 0.5 mg viz. 327 ± 30.52 and 312 ± 30.52 respectively, when compared to control eggs (only buffer ), i.e. 441±28.69 (Figure 7).

#### 3.7.2. Larvae stage

Hatched eggs went to larvae stage and lower number of larvae was observed at 1.5 mg, i.e. 12 ± 3 and other doses of 1.0 and 0.5 mg (32.6 ± 7.4 and 39 ± 7.4, respectively) showed significant differences when compared to control group (205 ± 22.74) (Figure 7).

#### 3.7.3. Pupae stage

Larvae, which survived and transformed to pupa stage, revealed maximum reduction in number i.e. 6 ± 3.67 at maximum dose of 1.5 mg when compared to control group (193 ± 19.59). Male and female pupae numbers were also recorded (3 ± 1.84 M, 3 ± 1.84 F), (10.6 ± 3.10 M, 10.6 ± 3.10 F), and (8.8 ±1 .56 M, 9.2 ± 1.46 F) for three treatments (1.5, 1.0, 0.5 mg, respectively). All doses showed great significant differences in reducing male and female pupae populations in comparison to control group of male and female pupae (96.6 ± 9.67 M, 96.4 ± 10.04 F, respectively) (Figure 7).

#### 3.7.4. Adult stage

At statistically significant difference of 0.05, concentrations of 1.0 mg and 0.5 mg showed significant differences (24 ± 6 and 30 ± 4.74) respectively, when compared to adults of control treatment i.e. 187 ± 20.89 while dose 1.5 mg showed great reduction of adults (4 ± 2.91) and also male and female numbers (2 ± 1.37, 2 ± 1.54, respectively) as compared to control group having maximum number of male (92 ± 11.24) and female (95 ± 9.74) adults. All doses having nonsignificantly mean pairwise differences from each other but showed significant differences from control group. The maximum dose, 1.5 mg, showed great effectiveness as compared to other doses (Figure 7).

### 3.8. Entomotoxicity against Sitophilus oryzae

**Figure 8:**
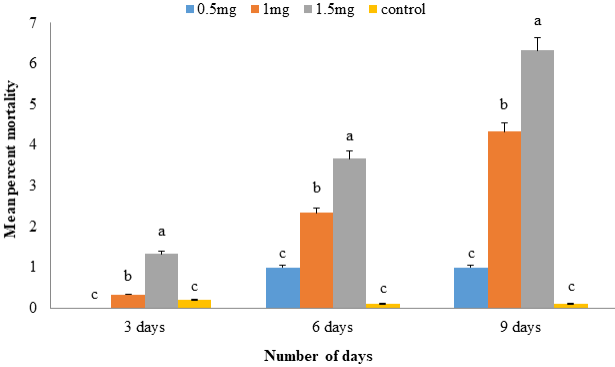
Mean percent mortality (±SE) of *S. oryzae* in response to three doses of *An*TI sprayed on rice kernels. Two treatments of *An*TI (1.0 and 1.5 mg) produced significant mortality in comparison to each other and as well as to control. However, the 0.5 mg treatment showed no significant mortality. Data were recorded after every third day up to maximum of nine days, until the adults mated and laid the eggs.

Adults of parent *S. oryzae* generation produced significant mean percent mortality in response to two treatments (1.0 and 1.5 mg) of *An*TI after 3, 6, and 9 days observation in comparison to control while the least concentration of 0.5 mg produced no significant mortality. After 9 days, 1.5 mg treatment showed maximum mean mortality of 6.33 ± 0.33 in comparison to control (Figure 8).

**Figure 9:**
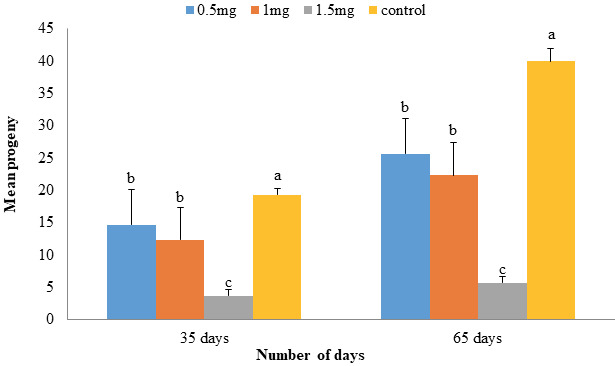
Two generation progeny of *S. oryzae* was studied in response to *An*TI. Effect of *An*TI on two generation (F1 & F2) progeny of *S. oryzae*; after 35 (first life cycle; F1) and 65 (second life cycle; F2) days respectively, was observed. *An*TI produced significant mortality of the F1 generation in comparison to control; however, F2 generation showed little mortality of the adults. Nevertheless, the highest treatment (1.5 mg) produced significant mortality as compared to rest of treatments.

After complete life cycle of *S. oryzae* F1 generation (35 days), the adults were counted for all four treatments. It was observed that three treatments of *An*TI produced significant reduction of adult population in comparison to control (Figure 9). Additionally, the highest dose of 1.5 mg was also found statistically significant from other doses which were nonsignificant with each other. After 65 days in second generation (F2), least rice weevils were observed on highest protein dose, i.e. 5.67 ± 0.88 followed by 1.0 and 0.5 mg as 22.33 ± 2.90 and 25.67 ± 1.76, respectively. Control produced maximum rice weevil progeny (40.0 ± 4.04) (Figure 9). Interestingly, it was observed that F2 generation showed less mortality in comparison to F1 generation on all three doses of *An*TI.

## 4. Discussion

This study was designed for the purification and thorough functional characterization of a trypsin inhibitor protein (*An*TI) which revealed strong antimicrobial and insecticidal potential. It exhibits substantial growth inhibition of human bacterial pathogens as well as of phytopathogenic fungal species. Similarly, strong entomotoxic activity of *An*TI has been observed against *Tribolium castaneum* and *Sitophilus oryzae* insects. 

The crude extract showed maximum concentration of *An*TI (290.95 mg) and purification fold of 1 which reduced in anion exchange (73.84 mg) and gel filtration (12.94 mg) chromatography with purification fold of 1.06 and 1.37, respectively. This increasing trend is in accordance with already reported findings of other researchers (Tripathi et al., 2014; de Silva Bezerra et al., 2016). On the other hand, specific activity (TIU) of crude extract (0.0411 × 105) was lower as compared to fractions collected from anion exchange (0.0433 × 105) and gel filtration (0.0564 × 105) column as already reported (Silva et al., 2015; Dias et al., 2017).

*An*TI was purified to homogeneity by using anion exchange column chromatography followed by size exclusion. A purified single band of 21 kDa under nonreduced condition was obtained which was cleaved in lower band of 17 kDa in the presence of *β*-mercaptoethanol. The lower band of 4 kDa was not visible on the gel. The analysis of multiple sequence alignment showed a highly homologous primary sequence of three proteins, i.e. *An*TI, *Ac*TI, and *Ec*TI. *Ac*TI is comprised of 175 residues and two chains named *α*- and *β*-chains which are linked by interchain disulfide bridge between Cys (133) and Cys (141). There are 136 and 39 residues in the *α*- and *β*-chains respectively. An intrachain disulfide linkage is also present in the *α*-chain linking Cys (40) with Cys (86). Under reduced condition, the two chains are separated from each other at residue Gly (136) into *α*- and *β*-chains as indicated by black arrow (Figure 2). The four half cysteines and N-terminii of *Ac*TI, *Acacia elata* and silk tree trypsin inhibitors are highly conserved (Kortt and Jermyn, 1981).

These inhibitor proteins are comprised of 3 repeating subdomains; each one constituted by 4 consecutive *β*-strands interconnected by loops. Two disulfide bridges are found in KTIs and none of them is located inside the active site; however, it is known that both disulfide linkages play an important role in the stabilization of the overall 3D structure (Aviles-Gaxiola et al., 2018). The two molecular structures shared more than 90% homology between their primary sequences and as a result a highly conserved molecular fold was observed between the two models *Ac*TI (magenta) & *Ec*TI (blue) (Figure 3B). 

Microbial pathogens have developed resistance against currently available antibiotics; which has triggered much interest in the isolation of antimicrobial proteins (Alasbahi and Melzig, 2008). The trypsin inhibitor protein from *Acacia nilotica* potently inhibits the growth of many pathogenic bacterial and fungal species and is therefore excellent candidate for the development of novel antimicrobial agents. The growth inhibition of different bacterial (*Staphylococcus aureus*,* Listeria monocytogenes*, *Escherichia coli*, and *Clavibactor michiganense*) and fungal species (*Rhizoctonia solani *and* Candida albicans*) by trypsin inhibitor proteins has already been reported (Kim et al., 2009). 

The quantitative calculation of the *An*TI inhibitory concentration responsible for 50% killing of the bacterial population was done through microtiter assay. A range of concentrations was tested to challenge the bacterial pathogens and 20 µg treatment was found to be effective for more than 50% growth inhibition of bacterial cells. *Albizia amara* trypsin inhibitor strongly inhibited the growths of *Pseudomonas aeruginosa* and *Bacillus subtilis* at concentrations of 16 and 32 μg of the protein in titer plate (Dabhade et al., 2016). Furthermore, 20 μg *An*TI concentration was tested against all pathogens through disc diffusion method which again produced strong zones of inhibition against all strains in comparison to negative control and 15 μg/disc *An*TI concentration. Trypsin inhibitors purified from the seeds of *Abelmoschus moschatus* had moderately inhibited the growth of *Pseudomonas syringae*,* Klebsiella pneumoniae, Pseudomonas aeruginosa*, and* Streptococcus pyogenes*, but showed a strong growth inhibition of *Bacillus cereus*, *Bacillus subtilis*,* Escherichia coli*,* Proteus vulgaris*, and* Streptococcus pneumoniae *at an inhibitor concentration of 50 μg (Dokka and Davuluri 2014). The inhibitors did not differentiate gram-negative bacteria from gram-positive bacteria in their antibacterial activity. Similarly, *Achyranthes aspera* trypsin inhibitor (AATI) significantly affected the growth of *Bacillus subtilis*,* Escherichia coli*, *Klebsiella pneumonia*, *Staphylococcus aureus*, and* Proteus vulgaris* at concentrations of 25 and 50 μg with considerable zones of inhibition (Konala et al., 2012). 

Likewise, 50 μg/well *An*TI concentration significantly reduced the conidial germination and further mycelial proliferation of *Fusarium oxysporum* and *Aspergillus niger*. *Albizia amara* trypsin inhibitor showed antifungal activity against *Alternaria alternata*,* Alternaria tenuissima*, and* Candida albicans* at concentrations of 32 and 64 μg/well (Dabhade et al., 2016). Similarly, microplate assay indicated strong inhibition of *Candida tropicalis* and *Candida buinensis* at 125 and 250 μg concentrations of *Inga laurina* trypsin Inhibitor (ILTI) (Macedo et al., 2016). To further strengthen our results, 50 μg *An*TI concentration was tested on petri dishes against two fungi and significant inhibition of fungal mycelia was observed in comparison to negative control. Three isoforms of serine protease inhibitors (ApTIA, ApTIB, ApTIC) of *Acacia plumosa* Lowe at concentrations of 100 μg inhibited the growth of* Aspergillus. niger*, *Colletotrichum* sp. P.10 and *Fusarium moniliforme *(Lopes et al., 2009). *Psoralea corylifolia* L. trypsin inhibitor inhibited the fungal growth of *Alternari brassicae*,* Aspergillus niger*,* Fusarium oxysporum*, and *Rhizoctonia cerealis* at 10 μM concentration (Yang et al., 2006). Comparative analysis of reported trypsin inhibitor concentrations emphasized clearly the strong potency of *An*TI against microbial pathogens and a good candidate for target drug development.

One way of inhibiting the microbial growth is the production of cell membrane channels by these inhibitors which lead to death of the cell because of the outflowing of cellular contents through these channels and this mode of action is different from that of antibiotics. However, the development of such channels is yet to be established (Konala et al., 2012). Similarly, plant trypsin inhibitors rely on the inhibition of serine proteases secreted by fungi for their nutritional needs and thus reducing the amino acid availability which is necessary for their growth and development (De Leo and Gallerani, 2002). Microbes also used extracellular proteases for gaining access into the host cell for the completion of their life cycle, but the development of stable enzyme–inhibitor complexes restricted their entry by interacting with the active site of the targeted protease which is incapable of enzymatic activity. Moreover, the option of such protease inhibitors entering into pathogens and inhibiting the functions of their intracellular proteases should also be considered (Duncan and In, 1991). 

Similarly, *An*TI showed strong entomotoxin activity against two most harmful stored grains insects *T. castaneum *and *S. oryzae*. A trypsin inhibitor from seeds of *Poincianella pyramidalis* (PpyTI) showed insecticidal activity against Mediterranean flourmoth (*Anagasta kuehniella*) with significant decrease in both larval weight as well as survival besides a larval stage extension. Biochemical analysis has demonstrated that the PpyTI affects the digestion process of insects through inhibition of their indigenous trypsin and chymotrypsin activities and such entomotoxin effect of PpyTI encourages further studies using this protein for insect pest control (Guimarães et al., 2015). A novel (PmTKI) produced strong deleterious effects on the mass and survival of *Ceratitis capitata* during larval development (Cruz et al., 2013). *Aedes aegypti* midgut proteases activity was reduced 50% by *Cassia leiandra* trypsin inhibitor (ClTI). Inhibitor promoted acute toxicity on the 3rd instar larvae of *Ae. aegypti*, with LC50 of 2.28 × 10−2 M. Moreover, it caused a 24-h delay of the larvae development and 44% mortality after 10 days of exposure (Dias et al., 2017). Apart from plant’s natural defense mechanism of using these PIs against such herbivores insects, such potent molecules (*An*TI) can also be used as synthetic formulations for the growth reduction of phytophagous insects by inhibiting their digestive proteases of midgut such as trypsin, chymotrypsin, and other similar enzymes; thus, leading to poor digestion and availability of essential amino acids resulting in starvation leading to death of the pest (Pandey et al., 2016). The molecular basis of enzyme-inhibitor complex has already been reported in which reactive loop (L5) is docked inside the active site of trypsin. The side chains of Arg 64 and Ile 65 of inhibitor occupies the S1 and S19 pockets of enzyme respectively. However, the reactive loop showed only minor adjustments after binding to trypsin molecule (Zhou et al., 2013). 

In summary, the present study confirms strong antimicrobial and insecticidal activities of *An*TI being a potential inhibitor of the indigenous proteinases of the target organisms. Hence, this inhibitor can be used as an alternative agent to synthetic antibiotics, fungicides, and pesticides.
